# Fisetin Inhibits Osteoclast Differentiation via Downregulation of p38 and c-Fos-NFATc1 Signaling Pathways

**DOI:** 10.1155/2012/810563

**Published:** 2012-09-12

**Authors:** Sik-Won Choi, Young-Jin Son, Jung-Mi Yun, Seong Hwan Kim

**Affiliations:** ^1^Laboratory of Translational Therapeutics, Pharmacology Research Center, Bio-Organic Science Division, Korea Research Institute of Chemical Technology, P.O. Box 107, Yuseong-gu, Daejeon 305-600, Republic of Korea; ^2^Department of Pharmacy, Sunchon National University, Suncheon 540-742, Republic of Korea; ^3^Department of Food and Nutrition, Kwangju Women's University, Gwangju 506-713, Republic of Korea

## Abstract

The prevention or therapeutic treatment of loss of bone mass is an important means of improving the quality of life for patients with disorders related to osteoclast-mediated bone loss. Fisetin, a flavonoid dietary ingredient found in the smoke tree (*Continus coggygria*), exhibits various biological activities, but its effect on osteoclast differentiation is unknown. In this study, fisetin dose-dependently inhibited the RANKL-induced osteoclast differentiation with downregulation of the activity or expression of p38, c-Fos, and NFATc1 signaling molecules. The p38/c-Fos/NFATc1-regulated expression of genes required for cell fusion and bone resorption, such as DC-STAMP and cathepsin K, was also inhibited by fisetin. Considering the rescue of fisetin's inhibitory action by NFATc1 over-expression, the cascade of p38-c-Fos-NFATc1 could be strongly involved in the inhibitory effect of fisetin on osteoclast differentiation. Furthermore, fisetin inhibited the bone-resorbing activity of mature osteoclasts. In conclusion, fisetin may be of use in the treatment of osteoclast-related disorders, including osteoporosis.

## 1. Introduction

The prevalence of osteoporosis is expected to increase as the population of elderly people increases. Osteoporosis is a common, systemic, degenerative skeletal disorder that is characterized by low bone mass (or bone mineral density) that can lead to an increased risk of fracture. Bone fractures result in serious problems including skeletal deformity, pain, increased mortality, and severe economic burden [1]; thus, the prevention or treatment of loss of bone mass and lifetime fracture is an important means of improving the quality of life of patients with disorders related to bone loss. 

Bone homeostasis is maintained by the balance between osteoclast-mediated bone resorption and osteoblast-mediated bone formation. Imbalances, caused most often by over-activated osteoclasts, can lead to loss of bone mass; this suggests that inhibiting osteoclast differentiation, resorptive activity, or both could be a promising strategy for treating patients with disorders such as osteoporosis. Current treatments for osteoporosis inhibit bone degradation through the use of bisphosphonates, but they are associated with unpleasant gastric side effects and a complicated dosing schedule. New antiresorptive agents should be both effective and safe for the long-term management of bone resorption-related disorders. 

Osteoclasts are bone-resorbing multinucleated cells derived from hematopoietic cells. Specifically, precursor cells of the monocyte macrophage lineage are differentiated into tartrate-resistant acid phosphatase (TRAP)-positive multinucleated osteoclasts in response to macrophage-colony stimulating factor (M-CSF) and receptor activator of nuclear factor-*κ*B ligand (RANKL). Especially, after RANKL binds to RANK, it stimulates the trimerization of its receptor, resulting in the activation of downstream signaling molecules such as MAP kinases [2]. RANKL-induced activation of MAP kinases further leads to the activation of transcription factors. RANKL strongly triggers the activation of two major transcription factors required for osteoclast differentiation, c-Fos and nuclear factor of activated T cells c1 (NFATc1), and these transcription factors play a critical role in the regulation of genes for osteoclast differentiation [3].

**Figure 1:**
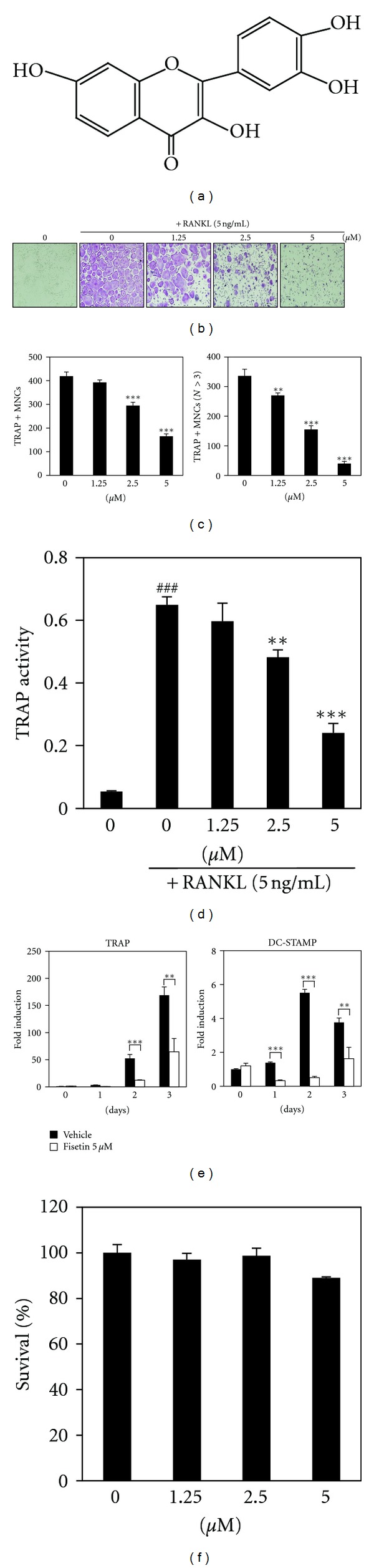
Fisetin inhibits RANKL-mediated osteoclast differentiation. (a) Structure of fisetin. (b) BMM cells were cultured for 4 days in the presence of RANKL (5 ng/mL) and M-CSF (30 ng/mL) with the vehicle (DMSO) or fisetin. Multinucleated osteoclasts were visualized to red-colored giant cells by TRAP staining. (c) Total TRAP-positive multinucleated osteoclasts (TRAP + MNCs; left graph) and TRAP + MNCs with more than 3 nuclei (*N* > 3; right graph) were counted. **P < 0.01; ***P < 0.001 (versus “the control”). (d) TRAP activity was measured. ^###^ *P* < 0.001 (versus “the negative control”). **P < 0.01; ***P < 0.001 (versus “the positive control”). (e) After pretreatment with the vehicle (DMSO) or fisetin (5 *μ*M) for 1 h, BMMs were treated with RANKL (5 ng/mL) for the indicated number of days, and then mRNA expression levels were analyzed by real-time PCR. *P < 0.05; **P < 0.01; ***P < 0.001 (versus “the vehicle control”). (f) Effect of fisetin on the viability of BMMs was evaluated by CCK-8 assay.

Natural products have historically yielded a variety of therapeutic agents. There have been many efforts to find natural products, including flavonoids that can prevent and treat osteoporosis while minimizing adverse side effects [4, 5]. Due to the abundance of flavonoids in dietary products and their beneficial pharmacological properties, flavonoids are of considerable interest as therapeutic agents as well as health food supplements. Fisetin (3,7,3′,4′-tetrahydroxyflavone; Figure 1(a)) is a flavonoid dietary ingredient found in the smoke tree (*Continus coggygria*); it is also widely distributed in fruits and vegetables, such as strawberries, apples, persimmons, grapes, onions, and cucumbers. A number of studies have investigated whether fisetin has various biological properties, such as antioxidant, antiangiogenic, antiinflammatory, antiaging, antiproliferative, antiapoptotic and antitumor activities [6–8]. To date, the effect of fisetin on osteoclast differentiation has not been studied, but its biological properties such as antiinflammatory activity might affect the osteoclast differentiation [9, 10]. Therefore, in this study, we investigated the effect of fisetin on osteoclast differentiation and possible molecular mechanisms of its action.

## 2. Materials and Methods 

### 2.1. Reagents and Antibodies

Penicillin, streptomycin, cell culture medium, and fetal bovine serum (FBS) were purchased from Invitrogen Life Technologies. Mouse soluble M-CSF and RANKL were purchased from R&D Systems. The CCK-8 assay kit was purchased from Dojindo Molecular Technologies. Antibodies against NFATc1, c-Fos, and actin were purchased from Santa Cruz Biotechnology. Antibodies against MAP kinases were purchased from Cell Signaling Technology. Fisetin was purchased from Sigma-Aldrich and dissolved in DMSO (Sigma-Aldrich).

### 2.2. Preparation of Osteoclast Precursor Cells

Bone marrow cells were obtained from 5-week-old male ICR mice by flushing femurs and tibias with *α*-MEM supplemented with antibiotics (100 units/mL penicillin and 100 *μ*g/mL streptomycin). Bone marrow cells were cultured in *α*-MEM supplemented with 10% FBS and M-CSF (10 ng/mL) for 1 day on culture dishes. Nonadherent bone marrow cells were plated on petri dishes and cultured for 3 days in the presence of M-CSF (30 ng/mL). After nonadherent cells were washed out, adherent cells were used as bone-marrow-derived macrophages (BMMs). 

### 2.3. Osteoclast Cell Culture and Osteoclast Differentiation

BMMs were maintained in *α*-MEM supplemented with 10% FBS, 100 units/mL penicillin, and 100 *μ*g/mL streptomycin. The medium was changed every 3 days in a humidified atmosphere of 5% CO_2_ at 37°C. To differentiate osteoclasts from BMMs, the BMMs (1 × 10^4^ cells/well in a 96-well plate) were cultured for 3-4 days with M-CSF (30 ng/mL) and RANKL (5 ng/mL). After 3-4 days, multinucleated osteoclasts were observed.

### 2.4. Cell Viability Assay

BMMs were plated in 96-well plates at a density of 1 × 10^4^ cells/well in triplicate. After treatment with M-CSF (30 ng/mL) and fisetin, cells were incubated for 3 days. Afterwards, cell viability was measured with the CCK-8 kit according to the manufacturer's protocol. 

### 2.5. Western Blot Analysis

Cultured cells were washed with ice-cold phosphate-buffered saline (PBS) and lysed in lysis buffer (50 mM Tris-HCl, 150 mM NaCl, 5 mM ethylenediaminetetraacetic acid (EDTA), 1% Triton X-100, 1 mM sodium fluoride, 1 mM sodium vanadate, and 1% deoxycholate) supplemented with protease inhibitors. Lysates were boiled in sodium dodecyl sulfate (SDS) sample buffer for 5 min and subjected to 10% or 12% SDS-polyacrylamide gel electrophoresis (PAGE) gels. After proteins were separated into gels, they transferred to a polyvinylidene difluoride membrane (Millipore). The membrane was then washed with TBST (10 mM Tris-HCl pH 7.5, 150 mM NaCl, and 0.1% Tween 20) and incubated in the blocking solution, TBST with 5% skim milk. The membrane was probed with the indicated primary antibody, washed three times for 30 min, incubated with secondary antibody conjugated to horseradish peroxidase for 2 h, and washed three times for 30 min. The membranes were then developed with SuperSignal West Femto Maximum Sensitivity Substrate (Pierce) using the LAS-3000 luminescent image analyzer (Fuji Photo Film Co., Ltd.). 

### 2.6. Real-Time PCR

Primers were chosen with the online Primer3 design program [11]. The primer sets used in this study are shown in Table 1. Total RNA was isolated with TRIzol reagent according to the manufacturer's protocol. First-strand cDNA was synthesized with 1 *μ*g of total RNA, 1 *μ*M oligo-dT_18_ primer, and 10 units of RNase inhibitor RNasin (Promega, WI) with the Omniscript RT kit (Qiagen) according to the manufacturer's protocol. SYBR green-based QPCR was performed with the Stratagene Mx3000P Real-Time PCR system and Brilliant SYBR Green Master Mix (Stratagene, CA), with the first-strand cDNA diluted 1 : 10 and 10 pmol of primers according to the manufacturer's protocol. The polymerase was activated at 95°C for 10 minutes, followed by 40 cycles of 94°C for 40 s (denaturation), 53°C for 40 s (annealing), and 72°C for 1 min (extension). This was followed by the generation of PCR-product-temperature-dissociation curves (also called melting curves) at 95°C for 1 min, 55°C for 30 s, and 95°C for 30 s. All reactions were run in triplicate, and data were analyzed by the 2^−ΔΔ*C*_*T*_^ method [12]. Glyceraldehyde-3-phosphate dehydrogenase (GAPDH) was used as an internal standard gene. The statistical significance was determined by Student's *t*-test with GAPDH-normalized 2^−ΔΔ*C*_*T*_^ values; differences were considered significant at *P* < 0.05.

### 2.7. TRAP Staining and Activity Assay

Multinucleated osteoclasts were fixed with 3.7% formalin for 10 min and permeabilized with 0.1% Triton X-100 for 10 min. The cells were stained with TRAP solution (Sigma-Aldrich). TRAP-positive cells were counted as multinucleated osteoclasts (nuclei ≥ 3) or TRAP-positive osteoclasts. To measure TRAP activity, multinucleated osteoclasts were fixed in 3.7% formalin for 5 min and permeabilized with 0.1% Triton X-100 for 10 min. The osteoclasts were treated with TRAP buffer (100 mM sodium citrate pH 5.0, 50 mM sodium tartrate) containing 3 mM *p*-nitrophenyl phosphate (Sigma-Aldrich) at 37°C for 5 min. Reaction mixtures in the wells were transferred into new plates containing an equal volume of 0.1 N NaOH, and optical density (OD) values were determined at 405 nm.

### 2.8. Retrovirus Preparation and Infection

Retrovirus packaging was described previously [13]. In brief, to isolate retrovirus, pMX-IRES-GFP (retrovirus vector, GFP; green fluorescent protein) and pMX containing constitutively active (CA)-NFATc1 were transiently transfected into Plat-E cells (platinum-E retrovirus packaging cell line; Cell Biolabs, Inc.) with Lipofectamine 2000 (Invitrogen) according to the manufacturer's protocol. Viral supernatant was collected from the culture medium 48 h after transfection. BMMs were incubated with viral supernatant in the presence of polybrene (10 *μ*g/mL) for 8 h. The infection efficiency of the retrovirus was determined by GFP expression and was always > 80%. After infection, BMMs were induced to differentiate in the presence of M-CSF (30 ng/mL) and RANKL (5 ng/mL) for 4 days.

### 2.9. Bone-Pit Formation Analysis

Mature osteoclasts were prepared by isolating osteoblasts from the calvariae of newborn mice by serial digestion in collagenase (Gibco), as previously described [14]. Bone marrow cells were isolated as described above. Osteoblasts and bone marrow cells were co-cultured on a collagen-coated 90-mm dish in the presence of 1*α*, 25-dihydroxyvitamin D_3_ (VitD_3_) and prostaglandin E_2_ (PGE_2_) for 6 days. Co-cultured cells were detached from the collagen-coated dishes, replated on BioCoat Osteologic MultiTest slides in 96-well plates, and treated with fisetin for 24 h. The cells on the BioCoat slides were stained for TRAP, and photographs were taken under a light microscope at 40x magnification. To observe resorption pits, cells on the BioCoat slides were washed with PBS and treated with 5% sodium hypochlorite for 5 min. After washing the plate with PBS buffer and drying, it was photographed under a light microscope. Quantification of resorbed areas was performed with the ImageJ program.

### 2.10. Statistical Analysis

All quantitative values are presented as mean ± SD. Each experiment was performed 3–5 times, and results from one representative experiment are shown. Statistical differences were analyzed using Student's *t-*test. A value of *P* < 0.05 was considered significant.

## 3. Results

### 3.1. Fisetin Suppresses RANKL-Induced Osteoclast Differentiation

To determine the effect of fisetin on RANKL-induced osteoclast differentiation, varying concentrations of fisetin were added to primary mouse BMM cultures in the presence of M-CSF (30 ng/mL) and RANKL (5 ng/mL) for 4 days. In the absence of fisetin, BMMs were shown to differentiate into mature TRAP-positive multinucleated osteoclasts, but in the presence of fisetin, the formation and number of TRAP-positive multinucleated cells were inhibited in a dose-dependent manner (Figures 1(b) and 1(c)); BMM differentiated into TRAP-positive multinucleated cells (red-color-stained giant cells in Figure 1(b)), but its formation was inhibited by fisetin. TRAP-positive multinucleated osteoclasts were counted in Figure 1(c). In addition, TRAP activity and mRNA expression were inhibited in the presence of fisetin (Figures 1(d) and 1(e)). Furthermore, the inhibitory effect of fisetin on osteoclast differentiation was confirmed by evaluating the mRNA expression level of DC-STAMP, which plays a role in cell fusion (Figure 1(e)); fisetin significantly inhibited the RANKL-induced mRNA expression of DC-STAMP. The presence of fisetin did not affect the survival of BMMs, indicating that the inhibitory effect of fisetin on osteoclast differentiation was not due to its cytotoxicity (Figure 1(f)). 

### 3.2. Fisetin Inhibits RANKL-Induced Phosphorylation of p38 and Expression of c-Fos and NFATc1

**Figure 2:**
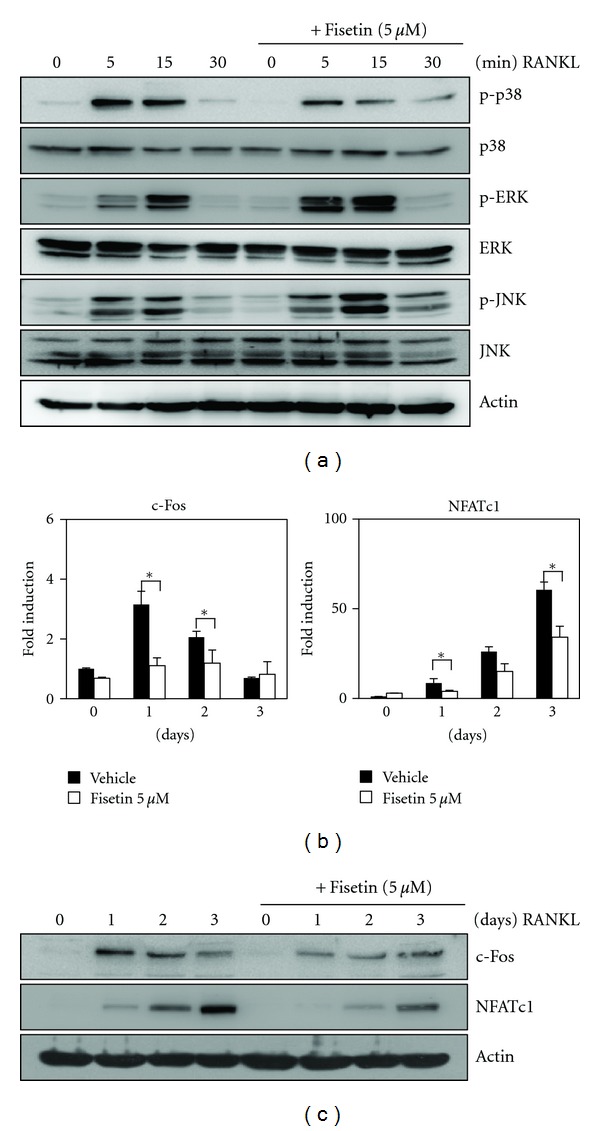
Fisetin inhibits RANKL-induced phosphorylation of p38 and expression of c-Fos and NFATc1. (a) BMMs were pretreated with vehicle or fisetin (5 *μ*M) for 1 h prior to RANKL stimulation (5 ng/mL) at indicated time periods. Then, protein expression levels were evaluated by western blot analysis. Actin was used as the internal control. (b) BMMs were stimulated with RANKL (5 ng/mL) and M-CSF (30 ng/mL) in the presence or absence of fisetin (5 *μ*M) for the indicated times. Then, total RNA was isolated from cells using TRIzol reagent, and mRNA expression levels were evaluated by real-time PCR. *P < 0.05 (versus “the vehicle control”). (c) Effects of fisetin on protein expression levels of c-Fos and NFATc1 were evaluated by western blot analysis. Actin was used as the internal control.

To elucidate the mechanism underlying the inhibition of RANKL-induced osteoclast differentiation by fisetin, we investigated the effect of fisetin on RANKL-induced early signaling pathways, including p38, JNK, and ERK. We found that fisetin only inhibited RANKL-induced phosphorylation of p38 (Figure 2(a)). In the process of osteoclast differentiation, RANKL-induced phosphorylation of p38 subsequently leads to the activation of early-stage and late-stage transcription factors, c-Fos and NFATc1, respectively [3, 15]. Therefore, we further examined the expression levels of c-Fos and NFATc1. Real-time PCR analysis revealed that fisetin strongly inhibited the RANKL-induced mRNA expression of both c-Fos and NFATc1 (Figure 2(b)). Additionally, western blot analysis showed that RANKL-induced protein expressions of c-Fos and NFATc1 were significantly suppressed by fisetin (Figure 2(c)). 

### 3.3. Ectopic Expression of NFATc1 Rescues Fisetin-Mediated Inhibition of Osteoclast Differentiation

**Figure 3:**
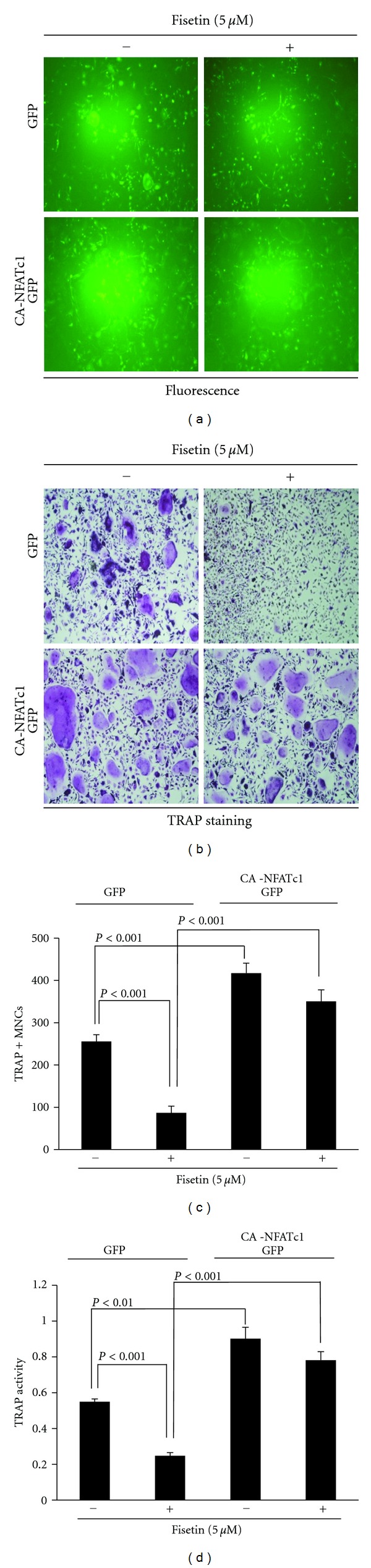
NFATc1 over-expression restores fisetin-mediated inhibition of osteoclast differentiation. (a) BMMs were infected with pMX-IRES-GFP (GFP) or pMX-IRES-CA-NFATc1-GFP (CA-NFATc1-GFP) for 8 h with polybrene (10 *μ*g/mL). Infected BMMs were cultured with M-CSF (30 ng/mL) and RANKL (5 ng) for 4 days in the presence or absence of fisetin (5 *μ*M). After 4 days, cells were fixed and GFP expression was visualized under a fluorescence microscope. (b) BMMs were infected with GFP or CA-NFATc1-GFP and then cells were cultured as described in (a). After 4 days, mature TRAP-positive multinucleated osteoclasts were visualized by TRAP staining. (c) TRAP-positive cells were counted as osteoclasts. (d) TRAP activity was measured at 405 nm.

When osteoclast differentiation is inhibited by downregulation of the p38-c-Fos-NFATc1 signaling axis, over-expression of NFATc1 restores osteoclast differentiation [13, 16]. Therefore, we evaluated whether the over-expression of NFATc1 could restore osteoclast differentiation that had been inhibited by fisetin. Considering GFP signaling, there was no difference in the infection yield between the control GFP and constitutively active (CA)-NFATc1-GFP plasmid (Figure 3(a)). Consistent with the aforementioned result, the formation of TRAP-positive multinucleated osteoclasts from BMM expressing the control GFP was strongly inhibited by fisetin (upper images in Figure 3(b)). However, even in the presence of fisetin, TRAP-positive multinucleated osteoclasts were formed from BMMs over-expressing NFATc1 (bottom images in Figure 3(b)), unlike those expressing the control GFP. The ameliorating effect of NFATc1 on the fisetin-mediated inhibition of osteoclast differentiation was also confirmed by counting the number of multinucleated osteoclasts (Figure 3(c)) and by measuring the activity of TRAP (Figure 3(d)). 

### 3.4. Fisetin Inhibits the Bone-Resorbing Activity of Mature Osteoclasts

**Figure 4:**
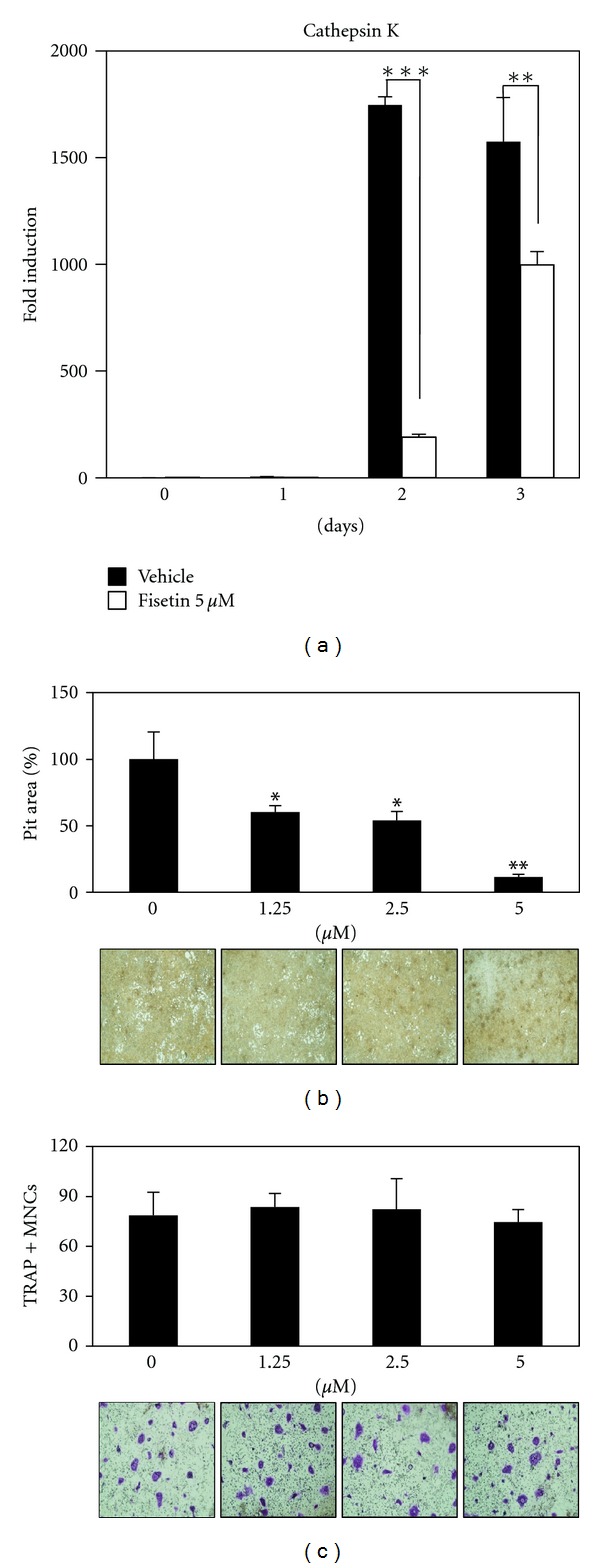
Fisetin inhibits osteoclastic bone resorption. (a) Effect of fisetin on mRNA expression of cathepsin K was analyzed by real-time PCR as described in Figure 1(e). **P < 0.01; ***P < 0.001 (versus “the vehicle control”). (b) Attached cells on BioCoat Osteologic MultiTest slides were removed and photographed under a light microscope (bottom images). Pit areas were quantified using ImageJ program (upper graph). **P < 0.01; ***P < 0.001 (versus “the control”). (c) Mature osteoclasts were plated on BioCoat slides and treated for 24 h with the indicated concentrations of fisetin. Cells were fixed, permeabilized, and stained with TRAP. TRAP-positive multinucleated cells were counted (upper graph) and photographed under a light microscope (bottom images).

Several natural products with inhibitory activity on osteoclast differentiation also inhibit the bone-resorbing activity of mature osteoclasts [17–19]. Therefore, we further evaluated whether fisetin has the potential to inhibit the bone-resorbing activity of mature osteoclasts. Fisetin inhibited the RANKL-induced mRNA expression of cathepsin K, which is an essential factor for bone resorption (Figure 4(a)). We next examined the antiresorptive activity of fisetin on synthetic carbonate apatite-coated plates. When mature osteoclasts were placed on carbonate apatite-coated plates and cultured in the presence or absence of various concentrations of fisetin for 24 h, fisetin dose-dependently inhibited the bone-resorbing activity of mature osteoclasts (Figure 4(b)); the resorbed areas (bright areas) on the slides were observed under a microscope (bottom images in Figure 4(b)) and measured as described in the Section 2 (upper graph in Figure 4(b)). To determine whether the inhibitory activity of fisetin on bone resorption might result from its potential to trigger the death of mature osteoclasts [9, 20], we counted the number of TRAP-positive multinucleated osteoclasts. As shown in Figure 4(c), fisetin did not affect the number of TRAP-positive multinucleated osteoclasts; there was no difference of the number of the red color TRAP-positive multinucleated osteoclasts between the control and fisetin-treated groups (bottom images in Figure 4(c)).

## 4. Discussion

In this study, fisetin was shown to dose-dependently inhibit osteoclast differentiation. The inhibitory effect of fisetin on osteoclast differentiation was also confirmed by evaluating the mRNA expression levels of TRAP and DC-STAMP. Considering that DC-STAMP has been shown to be essential for osteoclast fusion [21–23], fisetin might have the potential to inhibit this cell fusion. Cell fusion is a necessary event in the maturation of cells so that they can perform specific functions, such as bone resorption in the case of osteoclasts. 

The activation of MAP kinases is essential for osteoclast differentiation. Among MAP kinases, fisetin inhibited the RANKL-induced phosphorylation of p38. The involvement of the p38 signaling pathway in RANKL-induced osteoclast differentiation has been reported in several studies [24, 25]. Furthermore, the importance of p38 in inflammatory bone destruction has been suggested in several reports [26, 27], and it is considered to be a potential therapeutic target for inflammatory osteolysis [28]. Considering the antiinflammatory activity of fisetin [29] and its activity in preventing oxidative damage in osteoblasts [30], the potential antiresorptive property of fisetin could provide benefits for bone health. 

RANKL-induced activation of MAP kinases further leads to the activation of transcription factors such as c-Fos and NFATc1. Apparently, c-Fos and NFATc1 play a critical role in the regulation of genes for osteoclast differentiation. An important role for c-Fos in the process of osteoclast differentiation has been clarified in c-Fos knockout mice [31]; these mice had osteopetrosis due to osteoclast deficiency. Furthermore, NFATc1 has been shown to rescue osteoclastogenesis in cells lacking c-Fos [32–34]. These two transcription factors are also functionally linked together; c-Fos is essential for RANKL-mediated induction of NFATc1. c-Fos is expressed in the early stages of osteoclast differentiation, and it further regulates NFATc1 gene expression by binding to the promoter region of NFATc1. After NFATc1 is expressed in the middle or late stages of osteoclast differentiation, it subsequently regulates a number of osteoclast-specific genes, such as cathepsin K. 

In this study, fisetin inhibited the expression of c-Fos and NFATc1 at the transcriptional and translational levels. The induction of c-Fos and NFATc1 during RANKL-induced osteoclast differentiation is mediated by the p38 signaling pathway [3]; the inhibitory effect of fisetin on osteoclast differentiation could result from its potential ability to inhibit the p38-c-Fos-NFATc1 signaling axis. The recruitment of p38 and NFATc1 to target genes during osteoclast differentiation has been also reported [15], and the involvement of NFATc1 in fisetin-inhibited osteoclast differentiation was confirmed by the NFATc1 over-expression experiment performed in this study; the fisetin-induced inhibition of osteoclast differentiation was almost entirely rescued by NFATc1 induction. These results suggest that p38-c-Fos-NFATc1 signaling axis is involved in the inhibitory effect of fisetin on osteoclast differentiation. 

Cathepsin K is highly expressed in osteoclasts, and it is a well-known proteolytic enzyme that degrades the bone matrix [35, 36]. RANKL-induced cathepsin K gene expression has been shown to be cooperatively regulated by a combination of transcription factors, such as NFATc1 and p38 MAP kinase [37]. In this study, the presence of fisetin was associated with inhibition of the RANKL-induced mRNA expression of cathepsin K; furthermore, fisetin appeared to dose-dependently inhibit the bone-resorbing activity of mature osteoclasts. 

## 5. Conclusions

To our knowledge, this is the first study to report that fisetin has the potential to inhibit RANKL-induced osteoclast differentiation via attenuation of the RANKL-induced activation or expression of p38/c-Fos/NFATc1 signaling molecules. The decrease in signaling by p38/c-Fos/NFATc1 could consequently lead to a decrease in the expression of responsive genes required for cell fusion and bone resorption, such as DC-STAMP and cathepsin K. Furthermore, fisetin appeared to inhibit the bone-resorbing activity of mature osteoclasts. The potential antiresorptive property of fisetin could provide benefits for bone health and it may be of use in the treatment of osteoclast-related disorders including osteoporosis. 

## Figures and Tables

**Table 1:** Primer sequences used in this study.

Target gene	Forward primer (5′–3′)	Reverse primer (5′–3′)
TRAP	GATGACTTTGCCAGTCAGCA	ACATAGCCCACACCGTTCTC
DC-STAMP	CCAAGGAGTCGTCCATGATT	GGCTGCTTTGATCGTTTCTC
c-Fos	CCAGTCAAGAGCATCAGCAA	AAGTAGTGCAGCCCGGAGTA
NFATc1	GGGTCAGTGTGACCGAAGAT	GGAAGTCAGAAGTGGGTGGA
Cathepsin K	GGCCAACTCAAGAAGAAAAC	GTGCTTGCTTCCCTTCTGG
GAPDH	AACTTTGGCATTGTGGAAGG	ACACATTGGGGGTAGGAACA
